# Use of a sequential high throughput screening assay to identify novel inhibitors of the eukaryotic SRP-Sec61 targeting/translocation pathway

**DOI:** 10.1371/journal.pone.0208641

**Published:** 2018-12-13

**Authors:** Wolfgang Klein, Claudia Rutz, Jamina Eckhard, Becky Provinciael, Edgar Specker, Martin Neuenschwander, Gunnar Kleinau, Patrick Scheerer, Jens-Peter von Kries, Marc Nazaré, Kurt Vermeire, Ralf Schülein

**Affiliations:** 1 Leibniz-Forschungsinstitut für Molekulare Pharmakologie (FMP), Berlin, Germany; 2 KU Leuven, Department of Microbiology and Immunology, Rega Institute, Laboratory of Virology and Chemotherapy, Leuven, Belgium; 3 Charité—Universitätsmedizin Berlin, Campus Charité Mitte, Charité Centrum Grundlagenmedizin CC2, Institut für Medizinische Physik und Biophysik, Group Protein X-ray Crystallography & Signal Transduction, Berlin, Germany; University of Toronto, CANADA

## Abstract

The SRP-Sec61 targeting/translocation pathway of eukaryotic cells targets nascent protein chains to the membrane of the endoplasmic reticulum. Using this machinery, secretory proteins are translocated across this membrane whereas membrane proteins are integrated into the lipid bilayer. One of the key players of the pathway is the protein-conducting Sec61 (translocon) complex of the endoplasmic reticulum. The Sec61 complex has no enzymatic activity, is expressed only intracellularly and is difficult to purify and to reconstitute. Screening for small molecule inhibitors impairing its functions is thus notoriously difficult. Such inhibitors may not only be valuable tools for cell biology, they may also represent novel anti-tumor drugs. Here we have developed a two-step, sequential screening assay for inhibitors of the whole SRP-Sec61 targeting/translocation pathway which might include molecules affecting Sec61 complex functions. The resulting hit compounds were analyzed using a whole cell biosynthesis assay and a cell free transcription/translation/translocation assay. Using this methodology, we identified novel compounds inhibiting this pathway. Following structure-based back screening, one of these substances was analyzed in more detail and we could show that it indeed impairs translocation at the level of the Sec61 complex. A slightly modified methodology may be used in the future to screen for substances affecting SecYEG, the bacterial ortholog of the Sec61 complex in order to derive novel antibiotic drugs.

## Introduction

During the early stages of intracellular protein transport, nascent secretory and integral membrane proteins are synthesized at cytosolic ribosomes and must then be targeted to the membrane of the endoplasmic reticulum (ER), a process which is mediated by the SRP-Sec61 targeting/translocation pathway, a well described mechanism even at the structural level [1, 2]. Targeting of the proteins to the ER membrane is mediated by signal sequences. Two types of signal sequences are known: signal anchor sequences of integral membrane proteins, which are part of the mature proteins (usually transmembrane domain 1), and signal peptides of membrane and secretory proteins which are located at the extreme N terminus. Signal peptides are cleaved off during protein maturation. As soon as signal sequences of the nascent chains (NC) emerge at the translating ribosomes, they are bound by the signal recognition particle (SRP). Translation is slowed down and the resulting ribosome/NC/SRP complex is targeted to the heterodimeric SRP receptor at the ER membrane and from there to the Sec61 complex. The Sec61 complex contains the protein-conducting Sec61α channel subunit and the Sec61β and Sec61γ subunits. The NC is handed over to Sec61α, and the SRP/SRP receptor are disassembled by GTP hydrolysis, a complex reaction which was recently analyzed in more detail [3]. Following a tight interaction between the ribosome and the Sec61 complex, the signal sequence is involved in destabilization of the closed conformation of Sec61α causing the relocation of its plug domain (translocon gating). Translation accelerates, secretory proteins are translocated through the protein-conducting channel into the ER lumen whereas their signal peptides leave the protein-conducting channel at a lateral gate. Through this cleavage, secretory proteins are released into the ER lumen. Signal anchor sequences a well as the other transmembrane domains of integral membrane proteins are released through the lateral gate of the protein-conducting channel and become part of the ER membrane. The Sec61 complex is stably and transiently associated with supporting proteins such as the binding immunoglobulin protein (BiP, HSP70 chaperone family), Sec63 (HSP40 chaperone family), the translocating chain-associated membrane protein (TRAM), the translocon-associated protein complex (TRAP) and the oligosaccharyl transferase complex (OST).

For a pharmacological application, two types of inhibitors of the SRP-Sec61 targeting/translocation pathway may be important: those blocking the protein-conducting channel Sec61α in general thereby inhibiting the biosynthesis of all proteins using this pathway (broad ranging inhibitors, type A) and those blocking the function of individual signal sequences thereby inhibiting the biosynthesis of specific proteins (specific inhibitors, type B). Although a rather limited amount of studies addressed such inhibitors, some progress was made in the recent years.

Compounds targeting the Sec61 complex were identified more or less by chance during screens for inhibitors of the expression of specific proteins [4]. During a screen for inhibitors of the expression of cell adhesion molecules, the fungal compound HUN-7293, a cyclic heptadepsipeptide, was identified [5]. It was shown that HUN-7293 inhibits cotranslational translocation at the level of the Sec61 complex by a signal sequence-discriminative mechanism of action [6]. Similar results were reported for the cyclic heptadepsipeptide cotransin, which represents a simplified HUN-7293 derivative [7]. Originally, it was thought that these cyclodepsipeptides act rather selectively at the Sec61 complex affecting only the biosynthesis of a small group of proteins [6], [7] but more highly sensitive proteins were described in the following years [8–10]. A recent proteomic study for cotransin revealed, that at higher saturating concentrations, the biosynthesis of almost all secretory proteins was sensitive whereas the majority of integral membrane proteins remained cotransin-resistant [11]. The mechanism of action of cotransin and the related cyclodepsipeptides is still not completely clear. The cyclodepsipeptides mentioned above represent mixed type A/type B inhibitors (which means that they are selective rather than being broad range or specific inhibitors) which seems to preclude their pharmacological application.

Mycolactone, an immunosuppressive macrolide released by the human pathogen *Mycobacterium ulcerans* was recently shown to block transfer of secretory and integral membrane proteins at the Sec61 complex [12]. Mechanistically, it seems to perturb an interaction between the ribosome and Sec61α which is necessary for cotranslational translocation to progress [13]. Recent proteomic analyses, however, identified also mycolactone-resistant integral membrane proteins [14] suggesting that this compound has also a certain type of substrate selectivity at Sec61α which is however, much broader than that of the cyclodepsipeptides mentioned above. Another compound, namely Eeyarestatin I was shown to inhibit the ER associated degradation pathway ERAD) [15] but also acts at the level of the Sec61 complex where it prevents transfer of the NC from the targeting machinery to the Sec61 complex [16]. Moreover, it was shown that the compound inhibits retrograde transport pathways back to the ER in the case of at least some endocytosed proteins such as diphtheria toxin [17]. The natural compound apratoxin also inhibits Sec61α, most likely by binding to its lateral gate [18, 19]. Its actual selectivity, however, needs to be determined.

The available data for type B inhibitors of the SRP-Sec61 targeting/translocation pathway, i.e. those which interfere with specific signal sequences and consequently inhibit biosynthesis of specific proteins, are even more limited. The recently described compound cyclotriazadisulfonamide (CADA), however, binds specifically to the signal sequence of the human cluster of differentiation 4 protein (CD4), the cellular receptor for HIV. CADA inhibits CD4 biosynthesis at Sec61α, most likely by preventing a reorientation step of the signal sequence in the protein-conducting channel [20]. Only one additional protein, namely sortilin, was found to be sensitive in a proteomic study [21].

The setup of a high throughput screening assay for small molecule inhibitors of the SRP-Sec61 targeting/translocation pathway, in particular for the Sec61 complex, is very difficult since Sec61α has no enzymatic activity and is expressed only in the ER membrane. Here we set up a novel two step cellular screening assay allowing to identify both, type A and type B inhibitors (type B inhibitors could only be detected for the specific screening target, see below). With the help of this assay, we were able to identify novel compounds inhibiting cotranslational translocation at the Sec61 complex.

## Material and methods

### Plasmids and cloning

Standard DNA manipulations were carried out. The following G protein coupled receptors (GPCRs) were fused C-terminally with the enhanced green fluorescent protein (GFP) thereby replacing the stop codons using the vector plasmid pEGFP-N1 (Clontech, Mountain View, CA, USA): rat corticotropin-releasing factor receptor type 1 (CRF_1_R; residues M1-T413), rat corticotropin-releasing factor receptor type 2a (CRF_2(a)_R; residues M1-V411), human luteinizing hormone receptor (LHR; residues M1-C699), human protease-activated receptor 1 (PAR1; residues M1-T425), human thyrotropin receptor (TSHR; residues M1-L764), human endothelin B receptor (ET_B_R; residues M1-S442) and the human vasopressin V2 receptor (V_2_R; residues M1-K367). The resulting constructs were CRF_1_R.GFP, CRF_2(a)_R.GFP, LHR.GFP, PAR1.GFP, TSHR.GFP, ET_B_R.GFP and V_2_R.GFP respectively. The human water channel protein aquaporin 2 (AQP2; residues M1-K270) was also fused C-terminally with GFP using the same vector plasmid (resulting construct AQP2.GFP).

For the primary and secondary screens, stable transfected HEK 293 cell clones were derived expressing CRF_1_R.GFP and unfused GPP respectively under the control of the Tet-On promoter. To this end, both constructs were cloned into the pTRE-Tight vector of the Tet-On Advanced Inducible Gene Expression Systems (Clontech, Heidelberg, Germany). Establishing of the Tet-On system was carried out according to the supplier's recommendations.

For the fusion of the signal peptide of the CRF_1_R to the preprolactin marker, the previously described plasmid pGEMBP1 encoding bovine preprolactin (pPL) was used [22]. Similar to a previous study [21], inverse PCR-based site-directed mutagenesis was used to replace the pPL signal peptide in pGEMBP1 with the N-terminal 30 residues of the CRF_1_R (23 residues of signal peptide + 7 residues of mature CRF_1_R). The resulting construct was designated CRF_1_R-pPL.

### Cell culture

HEK 293 cells were cultured at 37°C and 5% CO2 in Dulbecco’s modified Eagle’s medium (DMEM, low glucose, GlutaMAX) containing 10% (v/v) fetal calf serum (FCS), penicillin (100 U/ml) and streptomycin (100 μg/ml).

### High-throughput fluorescence assay for the primary and secondary screens, concentration response validation, and data analysis

Using a microplate dispenser (EL406, Biotek), 30 μl of cell suspensions in DMEM were seeded into assay plates (black-wall with clear bottom cell-binding surface, 3683; Corning, NY, USA) in an initial density of 12,000 cells/well. The assay plates were incubated at 37°C, 5% CO_2_ in a humidity-controlled incubator for 24 h. Library compounds were dissolved to a final concentration of 10 mM in DMSO in columns 1 to 22 of a 384-well microtiter compound mother plate. Thereafter, 0.2 μl of compounds were transferred to a 384-well plate containing 40 μl DMEM (final compound concentration 50 μM) using a robotic liquid handler equipped with a 384-channel fixed-tip pipetting head (Freedom Evo, Tecan, Männedorf, Switzerland). 10 μl of this pre-diluted compound solutions were then transferred to the assay plates (using the same liquid handling equipment) and the plates were incubated for 1 h at 37°C to allow compound interaction with the target prior to induction of the GFP sensor construct. In a second microtiter plate containing DMEM, the inducer doxycyclin was added to a concentration of 5 μg/ml into columns 1 to 23, while column 24 received only medium. This scheme was applied to create positive controls in column 23 (induced cells without compound addition, 100% signal) and negative controls in column 24 (non-induced cells, 0% signal). Of this doxycyclin solution, 10 μl was transferred to the assay plates to induce GFP expression, giving the final assay conditions: 50 μl of cell growth medium, 10 μM of compound, and 1 μg/ml of doxycycline (where appropriate). Assay plates were incubated for another 48 h, and then the growth medium was removed to a residual volume of 10 μl using a microplate washer (Hydrospeed, Tecan) in order to reduce background fluorescence. GFP fluorescence was then measured in a microplate reader (Safire2, Tecan) with an excitation wavelength of 498 nm and an emission wavelength of 512 nm, with 5 nm bandpass each, and fluorescence reading from the bottom of the plate.

For concentration response validation of the compounds, 29 samples were rearranged from the compound store by placing 5 μl aliquots of 10 mM solutions onto columns 1 and 12 of a 384-well plate. Serial dilutions of the compounds were prepared by adding 5 μl DMSO to the compound samples, and transferring 5 μl of the diluted sample to the adjacent empty column. 11 consecutive serial 2-fold dilutions were prepared within the compound plate using a robotic liquid handler equipped with a 384-channel disposable tip head (tips were disposed after each pipetting step to ensure accuracy of the serial dilution). Compound samples were then diluted by transferring 1.6 μl to a 384-well plate containing 80 μl of DMEM, yielding 100 μM solutions. 10 μl of the pre-diluted solutions were transferred to the assay plates containing cells in 30 μl medium. Samples were incubated for 1 h at 37°C, and 10 μl of doxycycline pre-dilutions were added for target induction (final compound concentrations ranging from 0.02 μM to 20 μM). Fluorescence measurements were carried out as described above.

For the data analysis, percent activities were calculated for each measured sample by normalizing its readout to the medians of the plate control samples. To assess for statistical significance of a value, Z-scores were calculated for each sample [23]. For monitoring the integrity of the signal window, the Z’-factor was calculated for each plate using its positive and negative control samples [24]. Data were pre-processed (data normalization and initial quality control) using in-house software. Reports containing chemical structures and calculation of molecular properties were derived using Pipeline Pilot (BIOVIA, San Diego, CA, USA). IC_50_ values were determined by applying the four-parameter log-logistic function of the “drc” R-package for calculating concentration-response models [25]. Percent activity values were plotted against compound concentration, graphics were prepared using the R-statistics framework (R. C. Team, R Foundation for Statistical Computing, Vienna, Austria, 2016).

### Structure-based back screening and identification of FMP-401319 analogous compounds

The compounds were identified using the Tanimoto Molecular Similarity tool embedded in Pipeline Pilot and our in-house Database of Available Chemical Substances (DACS) containing ~38 million unique chemical structures coming from 43 different vendors (http://www.open-dacs.de/). According to a Tanimoto threshold of 0.6, 45 analogues of FMP-401319 were identified and thoroughly validated based on structure activity considerations. Compounds were purchased from commercial vendors and solubilized in DMSO.

### Whole cell flow cytometry biosynthesis inhibition assay

4.5 x 10^5^ HEK 293 cells were grown on 12-well plates for 24 h and transiently transfected with 1.2 μg plasmid DNA/well and polyethylenimine (PEI, Polysciences Europe GmbH, Eppelheim, Germany) according to the supplier’s recommendations. After 4.5 h of incubation, cells were treated for 19 h with compounds dissolved in DMSO (FMP-503533, 20 μM; FMP-214534 25 μM; FMP-401319 and its analogous substances, 25 μM; FMP-208236, 20 μM; FMP-214219, 10 μM) or cycloheximide (20 μg/ml) or DMSO alone (negative control). Variable concentrations of the compounds FMP-503533, FMP-214534, FMP-401319, FMP-208236, FMP-214219) were used due to the different IC_50_ values obtained for each compound in the screening assay. The final DMSO concentration in all samples was 1%. Cells were washed twice with PBS and the GFP fluorescence signals were quantified by flow cytometry using a FACSCalibur system (BD Biosciences, USA). Total fluorescence intensity of 1 x 10^4^ cells was analyzed for each sample using the BD CellQuest Pro software (BD Biosciences, USA). Normalization of the total amount of GFP fluorescence was realized by subtracting the background of non-transfected HEK 293 cells. The portion of the GFP fluorescence present at time t_0_ of compound treatment (proteins synthesized after transfection during the 4.5 h incubation time which may endure thereafter the compound incubation time) was eliminated by subtracting the value of cycloheximide-treated cells.

### Cell free transcription/translation and translocation assay

Truncated CRF_1_R-pPL NCs of 78 residues without a stop codon were first generated by standard PCR from pGEM plasmids, and subsequently transcribed *in vitro* using T7 RNA polymerase (RiboMAX system, Promega, Madison Wi, USA). Purified RNA was translated in rabbit reticulocyte lysate (Promega) in the presence of L-[35S]-methionine (PerkinElmer, Waltham, MA, USA). Translations were performed at 25°C for 20 min in the absence of microsomes. Pancreatic microsomes (RM) from sheep [26] were pre-treated with compound or DMSO control for 15 min at room temperature. Next, microsomes were added to the translation mixture and incubated for 10 min on ice, followed by 10 min incubation at 25°C. Release of the NCs from the targeted ribosome was induced by treatment with 2 mM puromycin (PU) for 7 min at 25°C. Equal aliquots of each sample were left untreated of were treated with proteinase K (PK, Roche, Basel, Switzerland). Digestion with PK was performed on ice for 30 min and quenched by the addition of phenylmethylsulfonyl fluoride (PMSF; Thermo Fisher Scientific, Waltham, MA, USA). Finally, 10 μl reaction mixtures were diluted with 300 μl low-salt buffer (80 mM KOAc, 2 mM Mg(OAc)_2_, 50 mM HEPES, pH 7.6), and radiolabeled proteins were centrifuged for 10 min at 21,382 x *g* and 4°C (Hettich 200R centrifuge with 2424-B rotor). The supernatant was removed, and the pellet was resuspended in 30 μl Laemmli sample buffer for analysis by SDS-PAGE. Quantitative analysis of translocation experiments was performed on a Cyclone Plus phosphor imager system (PerkinElmer) with accompanying software.

For the time-of-addition experiments, NCs were translated without microsomes as described above. In the mean-time, sheep pancreatic microsomes were pre-treated either with compound or with DMSO (control). After 20 min of translation, the translation mixture was split in 4 aliquots. Next, one aliquot received the compound pre-treated microsomes whereas the other 3 aliquots were given the control DMSO-treated microsomes. All samples were then incubated for 10 min on ice and 5 min at 25°C. The last 2 aliquots received either DMSO (control sample) or compound, and all samples were incubated for another 5 min at 25°C before treatment with PU. NCs were isolated by sedimentation at 4°C and pellets were dissolved in SDS sample buffer for analysis by SDS-PAGE as described above.

### Statistics

For the statistics of the screening procedures, data analysis and IC_50_ determination of the initial hit compounds, see above. Biosynthesis inhibition of the various membrane proteins by the compounds in the cellular assays and translocation inhibition in the cell free *in vitro* translation assays were analyzed using a One-way ANOVA test (Dunnett's Multiple Comparison Test). In the case of the cellular assay, mean values were calculated out of 3–10 independent experiments indicated in the figures ± SD. For the translocation inhibition experiments, mean values ± SD were calculated out of 2 or 3 independent experiments as listed in the S1 Table. P values: p ≤ 0.001 (***), p ≤ 0.01 (**), p ≤ 0.05 (*), p ≥ 0.1 (not significant, ns). GraphPad Prism Software, (La Jolla, CA, USA) was used in each case for statistics.

## Results and discussion

### Development and implementation of the high throughput screening strategy

**Fig 1:**
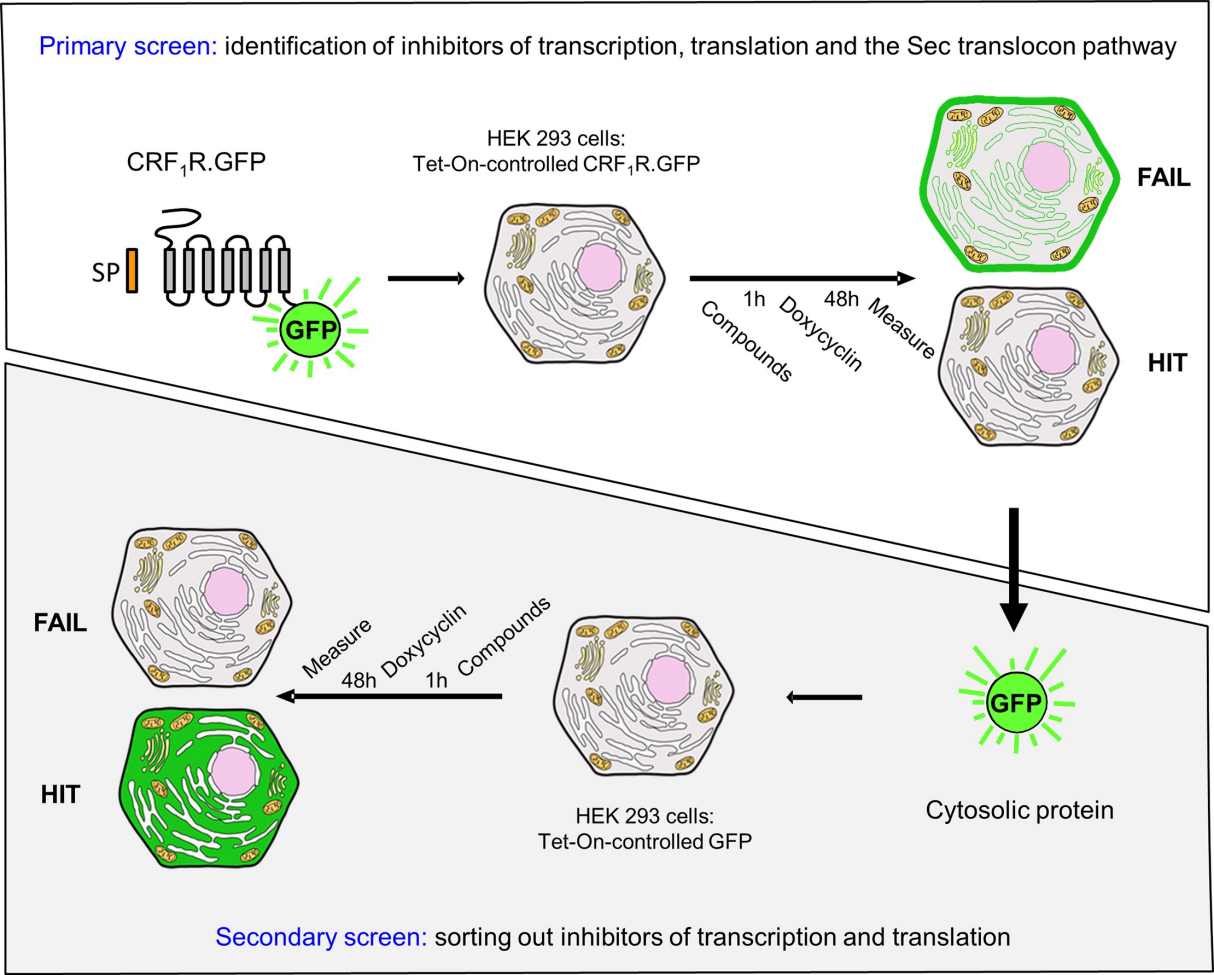
Scheme of the primary and the secondary screening procedures. In the primary screen (upper panel), construct CRF_1_R.GFP was used, a GFP-tagged GPCR possessing a cleavable signal peptide which uses the SRP-Sec61 targeting/translocation pathway. The secondary screen (lower panel) was performed with unfused, soluble GFP which does not use the SRP-Sec61 targeting/translocation pathway. See the text for details.

Taking the experimental obstacles mentioned above into account, we decided to establish a whole-cell screening approach involving two steps: first, inhibitors for transcription, translation and the SRP-Sec61 targeting/translocation pathway are selected (primary screen), then as a second step, inhibitors of transcription/translation are deselected (secondary screen) (Fig 1).

**Fig 2:**
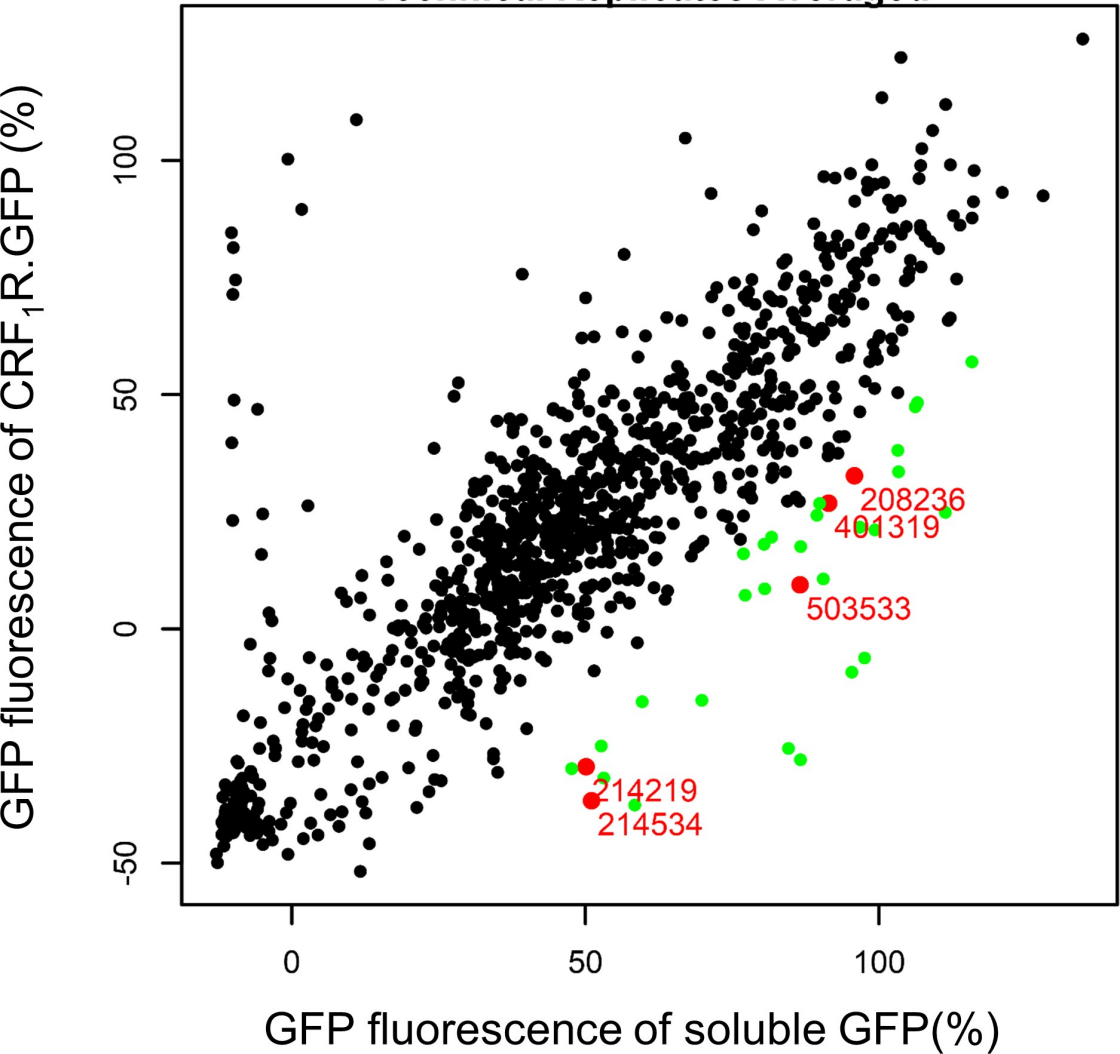
Schematic depiction of the results of the 1052 hit compounds under selection and deselection conditions. For each compound (dots), the relative GFP fluorescence of the screening target in the primary screen (CRF_1_R.GFP) was plotted against the relative GFP fluorescence of the target in the secondary screen (soluble, unfused GFP). Hit compounds are indicated in green, the 5 compounds used for further analysis are indicated in red (see below). Data represent mean values of three technical replicates (see S1 Fig for the replicates). 100% fluorescence = mean values of 16 induced plate control samples; 0% fluorescence = mean values of 16 non-induced plate control samples (plate control samples were without compound).

For the primary selection screen, we used the GFP-tagged CRF_1_R (CRF_1_R.GFP) as a target. The CRF_1_R was used, because it represents a well-studied GPCR which is highly expressed in the plasma membrane in comparison to other GPCRs. Moreover, it was experimentally confirmed that it passes the SRP-Sec61 targeting/translocation pathway by using its cleavable signal peptide [27]. Another advantage of taking a GPCR is that these receptors form the largest protein family in eukaryotic cells, which allows later on to analyze cross-selectivity of identified compounds on comparable protein targets. Since SRP binding to the N-terminal signal peptide of CRF_1_R.GFP should slow down or arrest translation, inhibitors of the SRP-Sec61 targeting/translocation pathway should decrease or even prevent CRF_1_R.GFP biosynthesis and consequently expression of the C-terminal GFP tag. The construct CRF_1_R.GFP was stably transfected in HEK 293 cells under the control of the Tet-On promoter. Without induction, the CRF_1_R showed almost no expression in these cells leading to a very good signal to noise ratio following induction which was an additional argument to take this receptor for our screening experiments. Cells grown on 384-well plates were pre-treated with the library compounds (10 μM each) for 1 h. The library encompassed 37,312 pre-selected substances with derived scaffolds based on the World Drug Index (WDI) (32,736) [28] and compound donations from academic research labs (4,576). Following pre-treatment of the cells with the compounds, CRF_1_R.GFP expression was induced and receptor expression was quantified by measuring the appearing GFP fluorescence signals. 1075 compounds significantly decreased receptor expression (z-score of the fluorescence signal < -3) in the primary screen, of which the 1052 most active compounds were re-picked onto new compound mother plates. The set of 1052 compounds was supposed to contain specific inhibitors for the SRP-Sec61 targeting/translocation pathway, inhibitors of transcription/translation, but also many stochastic false positives that would not confirm upon repetition of the measurement. A secondary deselection screen was thus performed using unfused, cytosolic GFP protein under the control of the Tet-on promoter as a target. This construct does not use the SRP-Sec61 targeting/translocation pathway and its expression depends only on transcription and translation. To identify compounds specific for the SRP-Sec61 targeting/translocation pathway, the 1052 compounds were re-measured under selection conditions in three technical replicates, and in parallel under deselection conditions also in three technical replicates at a concentration of 10 μM. 28 compounds reproducibly behaving as inhibitors in the selection screen, but not in the deselection screen, were considered to represent real hits. A graphical depiction of the activities of these 1052 hit compounds is shown in Fig 2.

**Fig 3:**
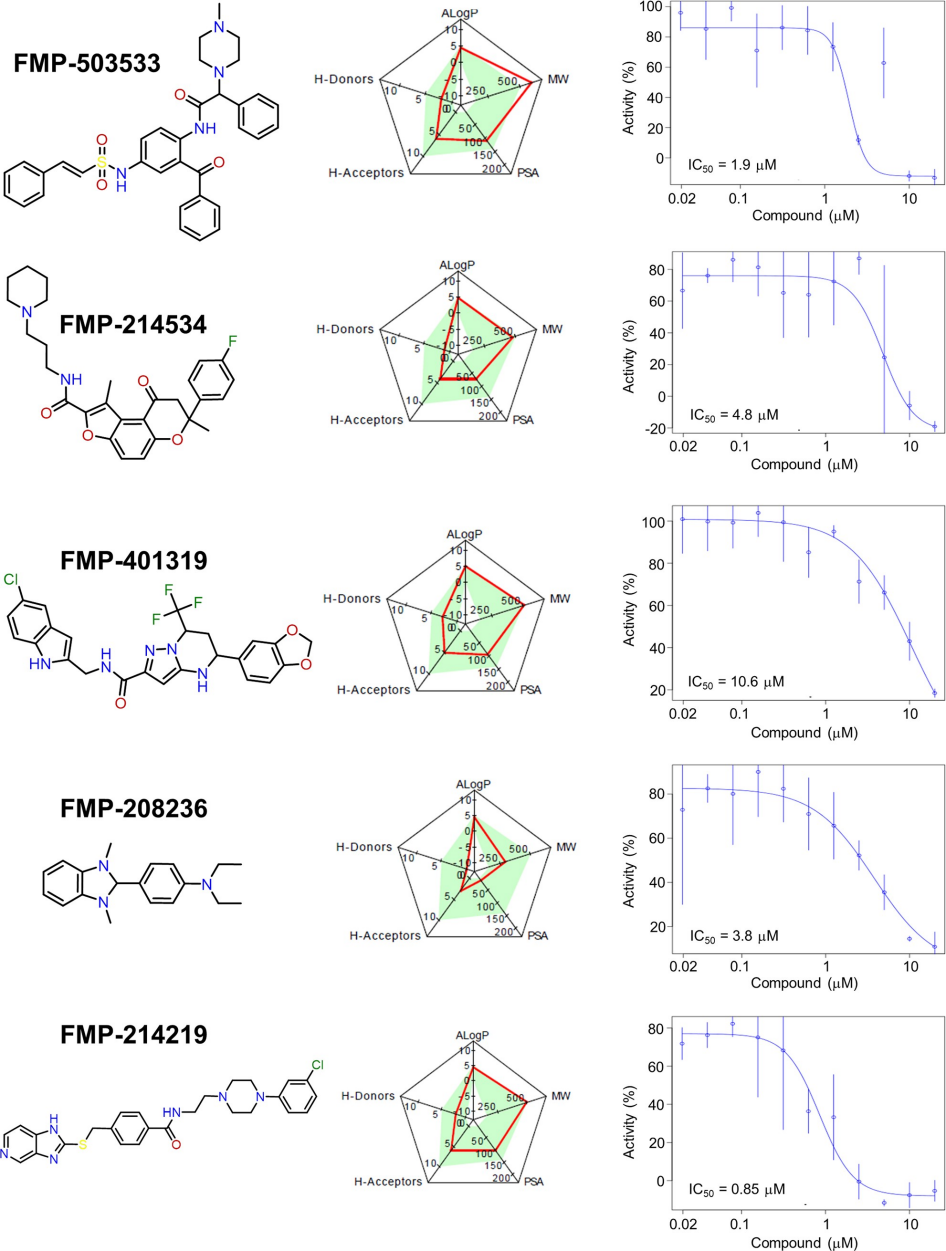
Chemical structures and properties of the compounds FMP-503533, FMP-214534, FMP-401319, FMP-208236 and FMP-214219. Left panels. Chemical structures of the compounds. Central panels. Rating of the compounds (red line) for their fulfilment of “Lipinski’s rule of five” for druggable compounds: H donors, H acceptors, hydrophobicity (ALogP), molecular mass (MW), and polar surface area (PSA). Calculation of the physicochemical properties was made using the SwissADME server (www.swissadme.ch). Right panels. Concentration response curves for the biosynthesis inhibition of the screening target CRF_1_R.GFP. The assay was carried out as in the primary screen using 384-well plates and different concentrations of the compounds. Expression of CRF_1_R.GFP was quantified fluorometrically *via* its GFP signals. The IC_50_ values are indicated. Data points are mean values of triplicates (±SD) and represent % of the maximal activity.

We assessed each compound for identity and purity by analytical liquid chromatography–mass spectrometry (LC-MS) experiments with a threshold of >90% purity. Using the same assay on 384-well plates with the 29 serially diluted compounds, the substances were rated with regard to their IC_50_ value for decreasing CRF_1_R.GFP expression (cut off = 15 μM). Moreover, we avoided compounds non-compliant to relevant descriptors of general drug-likeness such as the Lipinski`s “rule of five” (H donors, H acceptors, hydrophobicity, molecular mass) [29] and having a polar surface area (PSA) smaller than 140 Å^2^ [30] and Pan-assay interference compounds (PAINS; chemical compounds that are often false positive). In addition, we performed a database substructure search (ChEMBL, Chemical abstract, and our internal database) to evaluate potential biological activities and interference patterns of the identified chemotypes such as promiscuous binders to non-related protein targets. We focused on compounds that were neither over-represented as hits from in-house databases nor from curated online databases (Chemical abstract and ChEMBL) since frequent hit molecules could complicate downstream analyses. Finally, 5 hit structures possessing different chemotypes (FMP-503533, FMP-214534, FMP-401319, FMP-208236 and FMP-214219) remained for further validation analysis (Fig 3).

### Confirmation of the mode of action of the identified compounds by cellular and cell free assays

We next addressed the selectivity of the compounds for biosynthesis inhibition in a cellular assay. To this end, HEK 293 cells were transiently transfected with various GFP-tagged integral membrane proteins, namely AQP2.GFP, CRF_2(a)_R.GFP, ET_B_R.GFP, LHR.GFP, PAR1.GFP, TSHR.GFP, V_2_R.GFP and the original screening target CRF_1_R.GFP. The main interest of this study was to identify novel type A inhibitors of the Sec61 complex. Integral membrane proteins were chosen for this assay because the vast majority of them were resistant to mixed type A/type B inhibitors [11], such as cotransin, which we aimed to exclude from further analysis.

**Fig 4:**
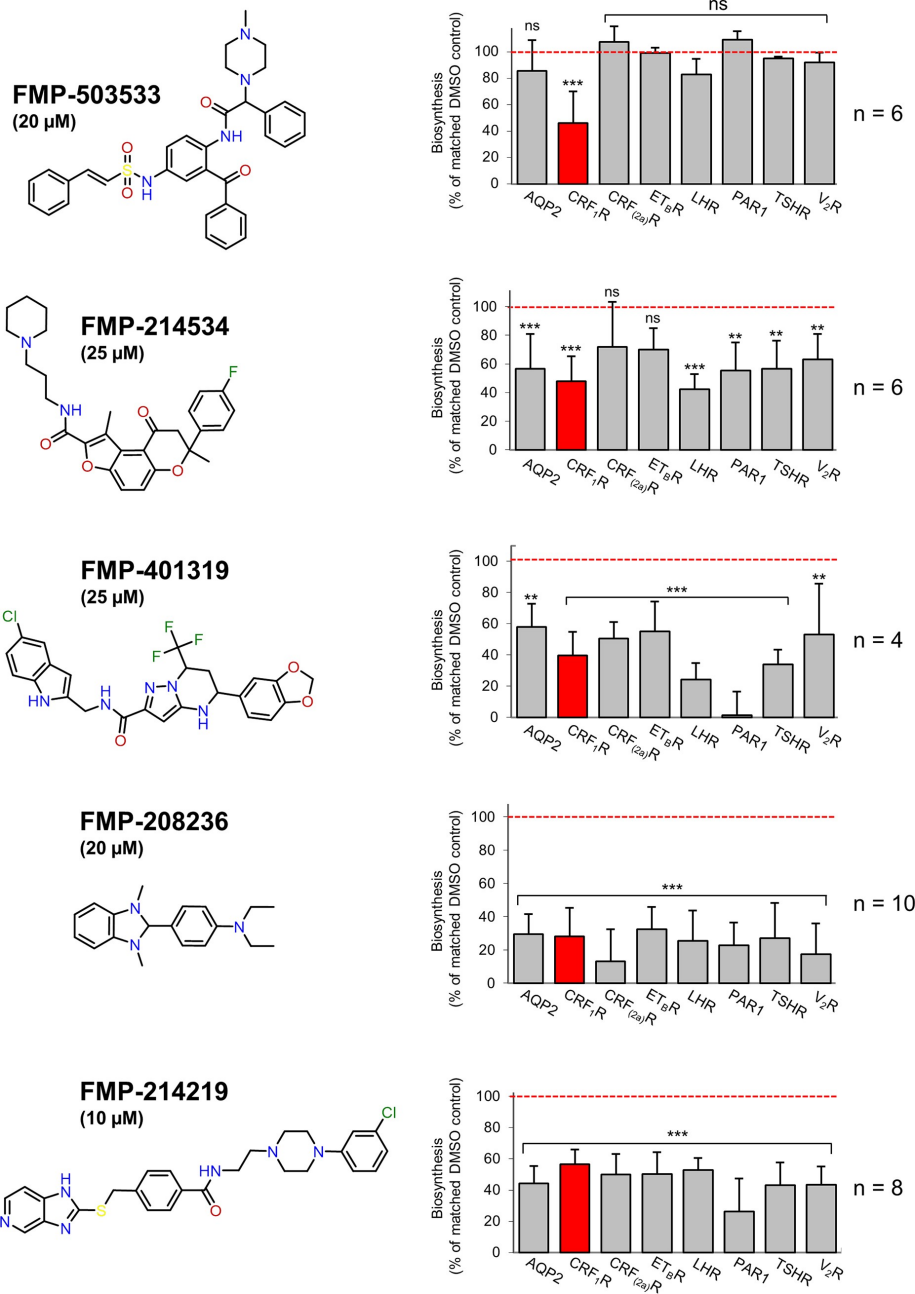
Whole cell assay: biosynthesis of various target proteins in HEK 293 cells treated with compounds FMP-503533, FMP-214534, FMP-401319, FMP-208236 and FMP-214219. To analyze activity and selectivity of the compounds, HEK 293 cells were transiently transfected with the original screening target CRF_1_R.GFP (red column) and various GFP-tagged integral membrane proteins. Cells were treated with the compounds (FMP-503533, 20 μM; FMP-214534, 25 μM; FMP-401319, 25 μM; FMP-208236, 20 μM; FMP-214219, 10 μM) or DMSO for 19 h and the total GFP fluorescence of the cells was analyzed using flow cytometry as a measure of biosynthesis. Note that compounds FMP-214534, FMP-401319, FMP-208236 and FMP-214219 affected the biosynthesis of all target proteins whereas compound FMP-503533 impaired only biosynthesis of the original screening target CRF_1_R.GFP. Columns represent mean values of biosynthesis (% of matched DMSO control, red line) calculated out of 3–10 independent experiments indicated in the individual panels ±SD. P values: p ≤ 0.001 (***), p ≤ 0.01 (**), p ≤ 0.05 (*), p ≥ 0.1 (not significant, ns).

Cells expressing the constructs were treated with the compounds or DMSO solvent or cycloheximide. After 19 h of incubation, the total GFP fluorescence of 1 x 10^4^ cells was analyzed using flow cytometry as a measure of membrane protein biosynthesis. To avoid falsification of the results by proteins already synthesized at t_0_ of compound treatment, cycloheximide values were subtracted. Compounds FMP-214534, FMP-401319, FMP-208236 and FMP-214219 inhibited biosynthesis of all target proteins albeit with different efficiencies (Fig 4). Taken together, these results are consistent with a general inhibition of the SRP-Sec61 targeting/translocation pathway of these compounds (type A inhibitor). Substance FMP-503533, in contrast, impaired only the biosynthesis of the original screening target CRF_1_R.GFP, suggesting that it is more selective and might e.g. interfere with the signal sequence of CRF_1_R.GFP (type B inhibitor).

**Fig 5:**
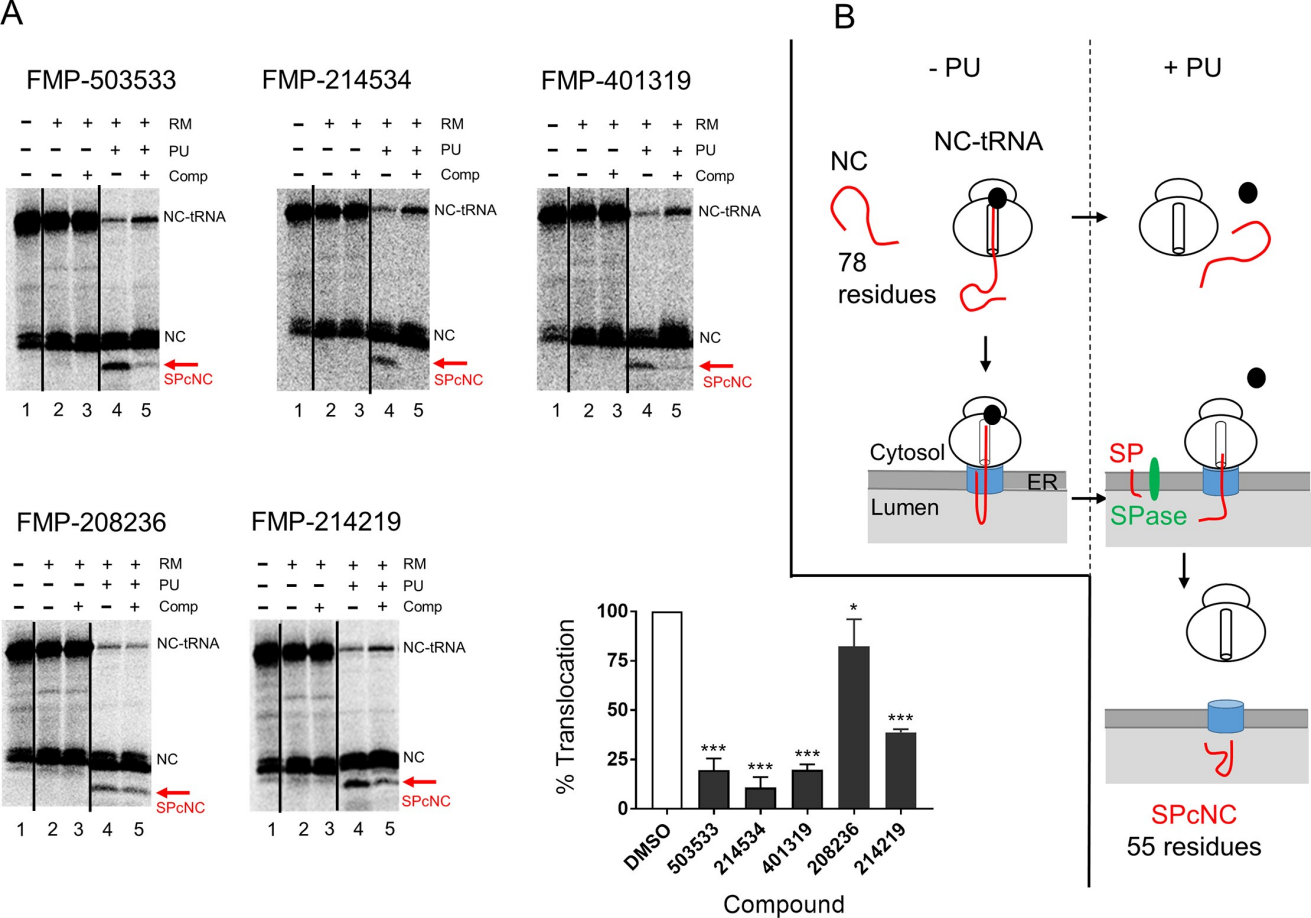
Cell free assay: activities of the compounds against the SRP-Sec61 targeting/translocation pathway. **(A)** Representative digital autoradiograms of the *in vitro* transcribed/translated and translocated CRF_1_R-pPL chimaera in the absence or presence of the indicated compounds (300 μM). NCs of 78 residues without a stop codon were transcribed/translated *in vitro* in the cell free rabbit reticulocyte lysate system. Because of an intact peptidyl-tRNA bond, NCs stay attached to the ribosome and appear as NC-tRNA complexes (lane 1, NC-tRNA). Due to spontaneous hydrolysis of the peptidyl-tRNA bond, a fraction of the [35S]-labeled peptides is visible as prematurely released NCs (lane 1, NC). Loaded ribosomes are subsequently mixed with RM (lanes 2–5, RM) to allow for ribosome docking onto the Sec61α protein-conducting channels. Note that the presence of RM also releases extra NCs from tRNA in an unproductive way. Finally, NCs are released from the ribosomes by addition of PU (lanes 4–5, PU), resulting in signal peptide-cleaved NCs that have been translocated into the ER lumen (SPcNC, 55 residues, red arrow). In the diagram on the lower right panel, each black bar represents the relative amount of translocated protein versus total protein (translocated and precursor) after compound treatment (lane 5), in comparison to the corresponding DMSO control (= 100% translocation; lane 4). Bars are mean ± SD.; n ≥ 2. See also S1 Table for the data. P values: p < 0.0001 (***), p ≤ 0.05 (*). **(B)** Schematic depiction of the cell free assay following *in vitro* transcription/translation of construct CRF_1_R-pPL and addition of RM. The effects of PU treatment (right panel) or leaving the sample untreated (left panel) on the NC-tRNAs are shown. See also (A) above for details. SP = signal peptide; SPase = signal peptidase.

We next performed a cell free assay to address the question whether the compounds indeed inhibit the SRP-Sec61 targeting/translocation pathway (Fig 5). The principle of the assay was as follows [20]: the signal sequence of the screening target CRF_1_R.GFP was fused N-terminally to the mature protein part of bovine pre-prolactin (pPL) in order to replace the native pre-prolactin signal sequence, and a PCR fragment without stop codon encoding for a 78-residue truncated NC of this CRF_1_R-pPL chimaera was synthesized (78mer; CRF_1_R-pPL see S2 Fig for the sequence and build-up of the construct). The purified PCR fragment was first transcribed *in vitro* using T7 RNA polymerase and subsequently translated in a cell free rabbit reticulocyte lysate mixture in the presence of radiolabeled [35S] methionine. Addition of pancreatic rough microsomal membranes (RM) resulted in docking of the ribosome-nascent chain complexes (RNCs) via the SRP receptor to the Sec61 complex and the NCs could engage Sec61α. However, due to the lack of the stop codon, the NCs were unable to complete translation/translocation and remained bound to the ribosomes *via* an intact peptidyl-tRNA bond (NC-tRNA). The NCs were thereafter released from the ribosomes (that are docked onto the protein-conducting Sec61α channel) by puromycin (PU) allowing not only full translocation into the ER lumen but also signal peptide cleavage by the signal peptidase (signal peptide-cleaved NCs; SPcNC). Using this assay, we could show that compounds FMP-503533, FMP-214534, FMP-401319 and to a slightly lesser extend FMP-214219 significantly inhibited translocation and processing of CRF_1_R-pPL (p < 0.0001) whereas compound FMP-208236 only had a minor effect on translocation inhibition, although with borderline significance (18% reduced translocation; p = 0.0467) (Fig 5A, the red arrow indicates the SPcNC fragment, i.e. the translocated CRF_1_R-pPL without signal peptide). We used a rather high concentration of the compounds in this assay (300 μM each), since previously published data for e.g. cotransin [7] and CADA [20] suggested that the IC_50_ values for biosynthesis inhibition at the Sec61 complex may be substantially higher in the cell free assay in comparison to a cellular assay. The reason for this discrepancy is not clear but it may be speculated that substantial amounts of these hydrophobic compounds dissolve in an unproductive way in the microsomal membranes which are present in excess.

These results show that compounds FMP-503533, FMP-214534, FMP-401319 and FMP-214219, indeed target the SRP-Sec61 targeting/translocation pathway. The mechanism of action of compound FMP-208236 remains elusive (although a general, non-Sec61 complex related cytotoxicity may be at play). On the other hand, the fact that this compound was unable to profoundly inhibit translocation and processing of CRF_1_R-pPL does not necessarily mean that this compound does not target the SRP-Sec61 targeting/translocation pathway. Since the components of the cell free assay need not to be synthesized and assembled as in whole cells, this method represents a reconstituted system. If, for example, compound FMP-208236 behaved as an assembly inhibitor of the Sec61 complex, such a function would not be detected.

### Substructure-based back screening and hit expansion of compound FMP-401319

**Fig 6:**
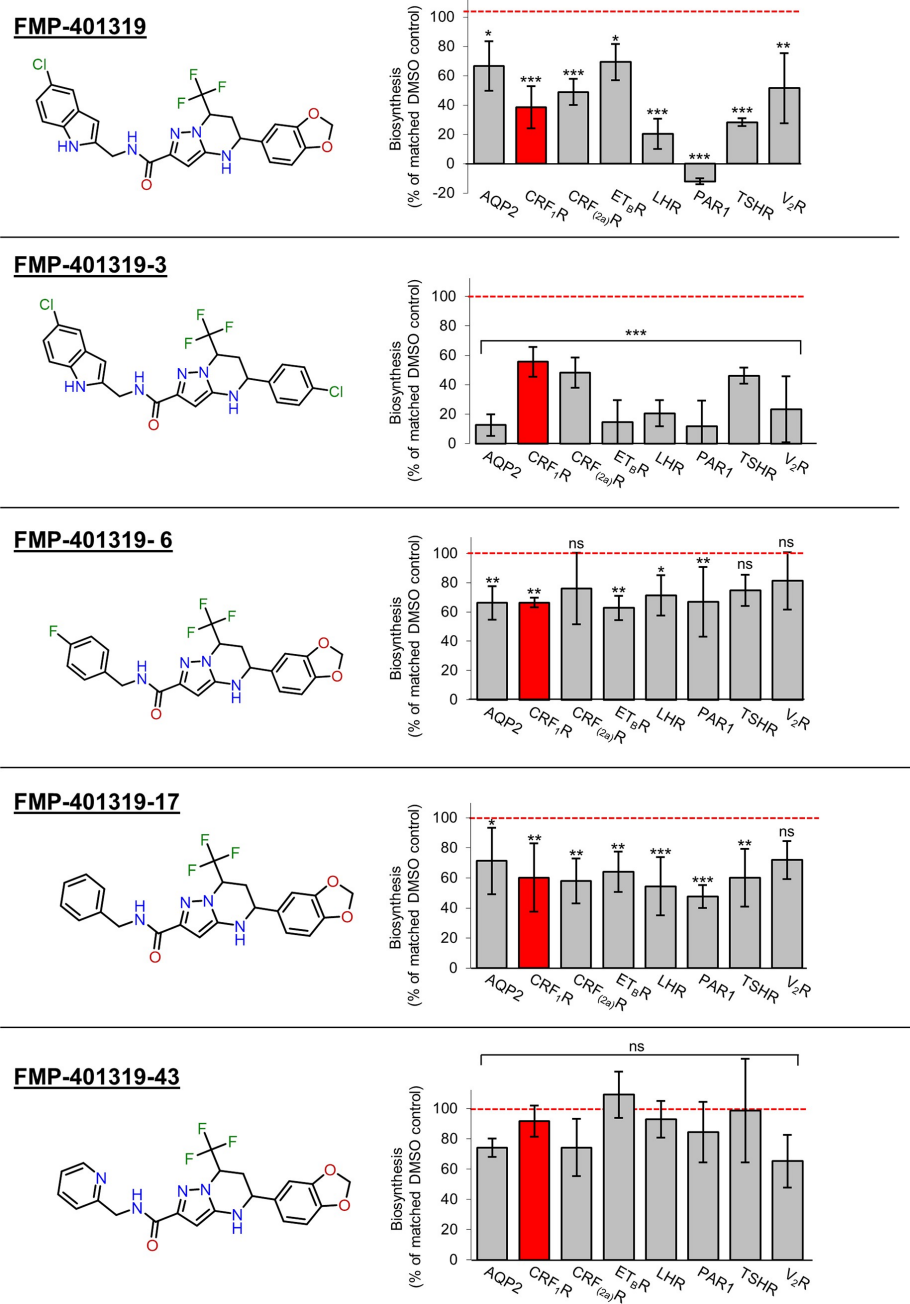
Substructure-based back screening of compound FMP-401319. Left panel. Structure of the compounds. Right panel. Commercially available derivatives of FMP-401319 (25 μM each) were tested for their inhibitory action in the whole cell biosynthesis assay as described in the legend of Fig 3. While some of the compounds showed a decreased activity (exemplary results: compounds FMP-401319-6, FMP-401319-17 and FMP-401319-43), one compound (FMP-401319-3) increased biosynthesis inhibition of some target proteins in comparison to FMP-401319. Columns represent mean values of biosynthesis (% of matched DMSO control, red line) calculated out of 5 independent experiments ±SD. P values: p ≤ 0.001 (***), p ≤ 0.01 (**), p ≤ 0.05 (*), p ≥ 0.1 (not significant, ns).

The next step was to look at the mechanism of action of the compounds in more detail, in particular to determine whether the compounds act at a pre-targeting step or at the level of the Sec61 complex itself. However, as the number of active cluster members and an existing structure activity relationship (SAR) within the same chemotype can be considered as a further evidence supporting the validity of a hit compound, we first performed a substructure based back-screening for additional commercially available substances around the compounds FMP-503533, FMP-214534 and FMP-401319. In the case of compound FMP-401319, a large cluster of similar compounds bearing the pyrazolo[1,5-a]pyrimidine scaffold was commercially available. We tested 45 analogues by the whole cell selectivity assay described above. While some of the compounds showed a decreased activity (e.g. compounds FMP-401319-6, FMP-401319-17 and FMP-401319-43), one compound (FMP-401319-3) exhibited a significantly enhanced biosynthesis inhibition of some target proteins (AQP2, ET_B_R, V_2_R) in comparison to the parental hit FMP-401319 (see Fig 6 for exemplary results). Of note and for an unknown reason, the PAR1 seems to be most sensitive for FMP-401319-3 and also for the parental compound FMP-401319 (see Fig 4). In the case of PAR1, the protein sequence downstream (thus, C-terminal) to the signal peptide cleavage site of is auto-proteolytically released as a peptide called parstatin, following receptor activation by thrombin [31]. Thus, the sequence adjacent to the signal peptide of the PAR1 may not be simply exchangeable as it has to match functionally to the preceding signal peptide to form a loop during Sec61α gating, like it has been shown previously for the signal peptides and the downstream C-terminal sequences of other membrane proteins [32, 33]. It remains to be determined whether such a hypothetic functional match influences sensitivity in this case.

**Fig 7:**
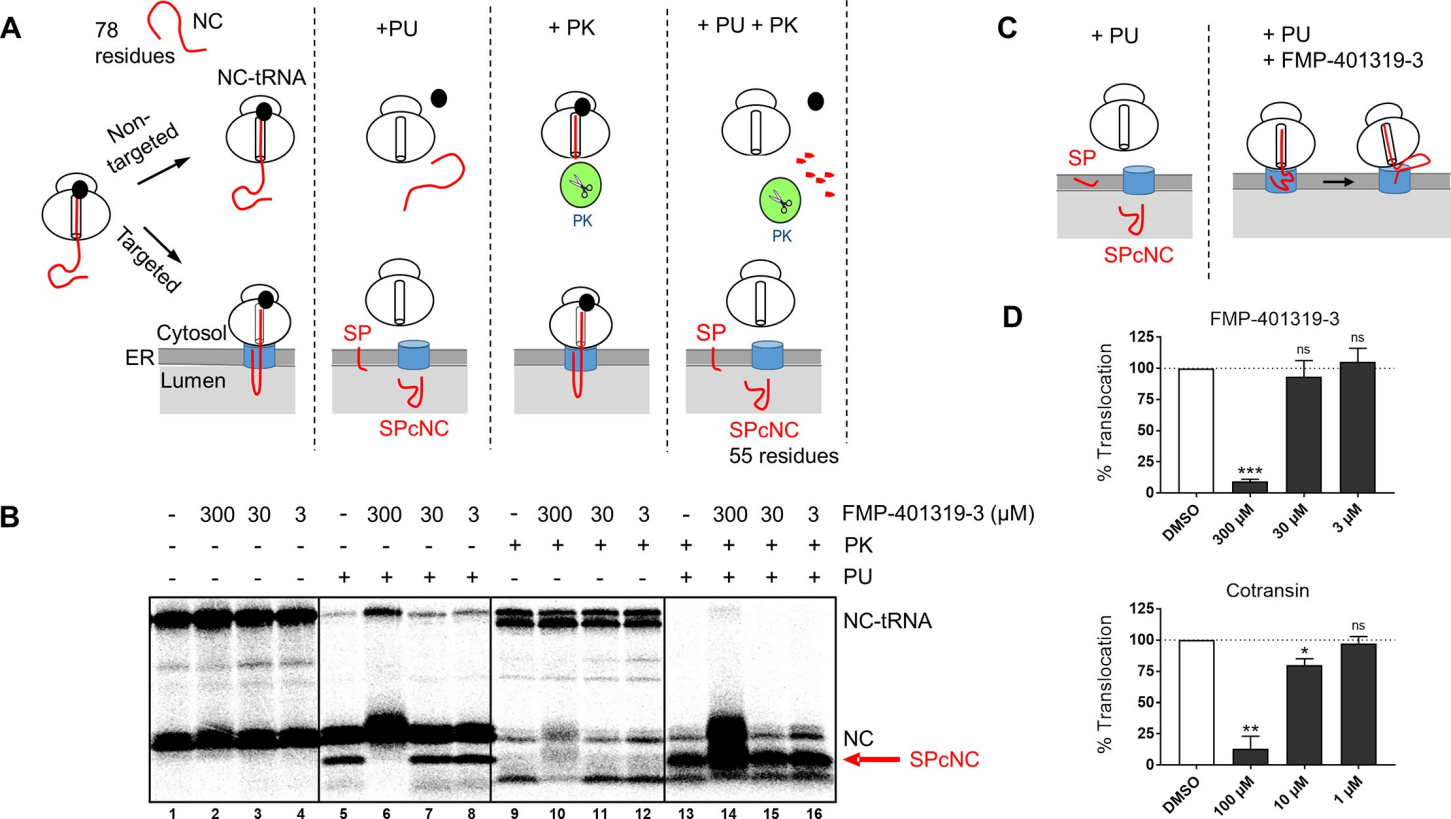
Compound FMP-401319-3 inhibits cotranslational translocation of CRF_1_R-pPL concentration-dependently at the level of the Sec61 complex. **(A)** Schematic depiction of the effects of PU, PK and a PU/PK combination on targeted and non-targeted ribosomes containing CRF_1_R-pPL NC-tRNAs in the cell free transcripition/translation/translocation assay. See text for details. The individual panels of Fig 7A refer to the autoradiogram shown in 7B. **(B)** Representative digital autoradiogram of the translated and translocated CRF_1_R-pPL chimaera in the absence or presence of different concentrations of compound FMP-401319-3 (3 μM, 30 μM, 300 μM), as described in the legend to Fig 5. All samples are with RM. Equal aliquots of the translated material were treated with PK (lanes 9–16, PK) or left untreated (lanes 1–8). SPcNCs that have been translocated into the ER lumen (red arrow) are protected inside the microsomal vesicles and thus PK-resistant. Note that at 300 μM of FMP-401319-3, most peptide chains could be rescued in the intact NC form after PK treatment (lane 14). **(C)** Interpretation of the results following FMP-401319-3 treatment. Without compound treatment (left panel), the NC is fully translocated, the signal peptide is cleaved-off and the SPcNC fragment appears. Treatment with FMP-401319-3 (right panel), however, interferes with a step before the growing peptide chain has been translocated and reached the luminal side of the ER. Some NC may be positioned between ribosome exit tunnel and the cytosolic side of the Sec61 complex in such a way that some parts are exposed to the cytosol and accessible in the experiment to PK. **(D)** Quantification of the data shown in Fig 7B and from 2 additional, independent experiments. Graph in upper panel shows the percentage of translocation for different concentrations of compound FMP-401319-3, similar as explained in the legend to Fig 5A. Bars are mean of three digital autoradiograms ± SD; n = 3. See also S1 Table for the data. Lower panel displays the results from equivalent experiments with the archetypical mixed type A/type B inhibitor cotransin (a representative digital autoradiogram is given in S3 Fig). It was previously shown that the signal peptide of the CRF_1_R is cotransin-sensitive [7]. Bars are mean of three digital autoradiograms ± SD; n = 3. See also S1 Table for the data.

This experiment also allows a first insight into the underlying molecular recognition and structure activity relationship of FMP-401319. For example, substitution of the benzo-1,3-dioxolan group (eastern half of the molecule) by a chlorophenyl group led to a better inhibitory activity (FMP-401319-3) while substitution of the 5-chloro-indol group (western half of the molecule) decreased inhibitory activity of the compound. This indicates that both distal moieties at the pyrazolo[1,5-a]pyrimidine scaffold play an important role for its efficacy. Moreover, we analyzed the compound in the cell free assay described above and found a slightly increased inhibition of CRF_1_R-pPL translocation in comparison to the original compound FMP-401319. The absolute IC_50_ values for translocation inhibition were 139 μM for FMP-401319 and 98 μM for FMP-401319-3 (see also Fig 7 and S1 Table for the data). The IC_50_ values were obtained by calculating the amount of translocated protein versus total protein (translocated and precursor), in comparison to the DMSO control (= 100% translocation). The IC_50_ value determined for FMP-401319 in the whole cell experiment (10.6 μM; see Fig 3) was roughly tenfold higher in the cell free assay. As already mentioned, a similar discrepancy between the IC_50_ values in whole cell and cell free assays was observed in other studies for inhibitors acting at the level of the Sec61 complex, e.g. for the more or less substrate-selective compound cotransin [7] or CADA which inhibits highly selective the translocation of human CD4 [20]. Due to the improved activity of FMP-401319-3, we focused for the subsequent studies on this compound.

### Compound FMP-401319-3 acts in a post targeting step at the level of the Sec61 complex

To determine whether a pre- or post-targeting step is affected by compound FMP-401319-3, we repeated the cell free assay described above but equal aliquots of the translated/translocated material were left untreated or were exposed to proteinase K (PK, see Fig 7A for a scheme of the experiments). Another aim was to obtain additional information on how the ribosome docks onto Sec61α and how the NC is inserted into the protein-conducting channel. NCs of 78 residues are bound to the ribosome as peptidyl-tRNAs with about 30 residues buried inside the ribosome tunnel. PK treatment of these free, non-Sec61 complex-bound ribosome nascent chains (RNCs) will degrade the N-terminal part of the chain that is exposed to the cytosolic compartment (about 48 residues), generating a slightly faster migrating protein band on the gel (directly underneath the NC-tRNA species) that correspond with the unproductive, non-targeted RNCs (Fig 7B, lanes 9–12). If well-targeted, NCs will be shielded from exogenous protease because of a tight interaction between the ribosome and the protein-conducting channel after transfer from SRP. If so, they will appear as intact RNCs after PK exposure. Because equal PK-protected NC-tRNA bands were observed in DMSO and FMP-401319-3 treated samples (Fig 7B, lanes 9–12, top band), we can rule out an inhibitory activity of this compound on targeting and transfer of the NCs of CRF_1_R-pPL to the Sec61 complex. However, addition of PU to the samples in order to release the NCs from the ribosome did not result in translocation and signal peptide cleavage (SPcNC fragment; red arrow) of the chains when FMP-401319-3 was present in a higher concentration (Fig 7B, lane 6 compared to lane 5). Compound FMP-401319-3 kept most peptide chains in the non-cleaved form as intact NC (Fig 7B, lane 6), that were also protected from PK degradation (lane 14), indicating that FMP-401319-3 inhibits the cotranslational translocation of CRF_1_R-pPL by holding the NC inside the protein-conducting channel (see Fig 7C for an interpretation of the results). The appearance of additional peptide fragments that are PK resistant (Fig 7B, lane 14) suggest that at least some NCs in the presence of FMP-401319-3 may be positioned between ribosome exit tunnel and the cytosolic side of the Sec61 complex in such a way that some parts are exposed to the cytosol and accessible to exogenous protease (see Fig 7C for an interpretation of the results). A quantification of the translocated amount of CRF1R-pPL under the influence of compound FMP-401319-3 at the different concentrations is shown in Fig 7D. In comparison to the archetypic mixed type A/type B Sec61 complex inhibitor cotransin, the compound has an approximately 3-fold lower potency (it was previously shown that the signal peptide of the CRF_1_R is cotransin-sensitive [7]).

**Fig 8:**
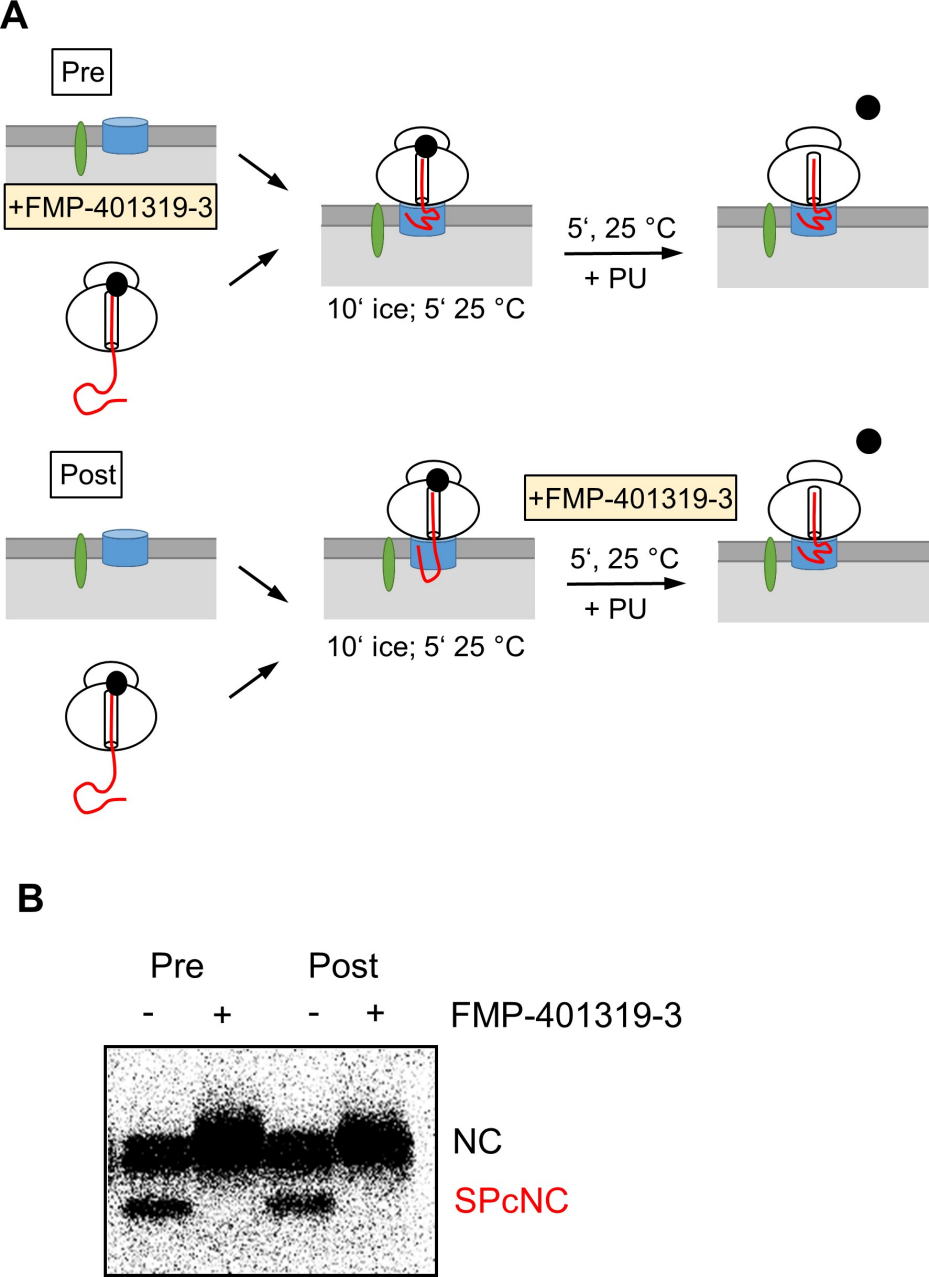
Compound FMP-401319-3 inhibits cotranslational translocation of CRF_1_R-pPL in a post targeting step. **(A)** Schematic depiction of the experiment. CRF_1_R-pPL NCs of 78 residues were translated in the absence of microsomes before administration to the RM for targeting. NCs were left untreated or were treated with FMP-401319-3, either applied to RM for pretreatment (pre), or applied to the RNC/RM mixture 15 minutes after initiation of targeting but before PU release (post). **(B)** Representative autoradiogram of the translated and translocated CRF_1_R-pPL chimaera in the absence (-) or presence (+) of compound FMP-401319-3 (300 μM). Pre = drug treatment before targeting. Post = drug treatment initiated 15 minutes (i.e., 10 + 5) after targeting. The NC and the SPcNC fragments are indicated.

Consistent with the results shown above, we could also postpone the administration of compound FMP-401319-3 to the RM until targeting was completed. A similar inhibitory effect on the translocation of CRF_1_R-pPL 78-mers was obtained when the microsomes were pretreated with the compound (i.e. drug treatment before targeting) as when the compound was applied to the RNC/RM mixture 15 minutes after initiation of targeting (Fig 8).

Whereas these results show that FMP-401319-3 blocks cotranslational translocation at the level of the Sec61 complex, the detailed mechanism of action of this compound remains elusive. Our data suggest that compound FMP-401319-3 interferes with a step after the targeting and transfer of the NCs to the Sec61 complex, but before the growing peptide chain has been translocated and has reached the luminal side of the ER in a looped form where signal peptide cleavage can occur. It should be stressed, however, that we do not know whether compound FMP-401319-3 binds directly to the Sec61 complex or has an indirect mechanism of action. Based on the X ray structure of the archaebacterial SecYEG [34] (S4A Fig) and the canine cryo-EM structure of the Sec61α [35] (S4B Fig), we constructed a structure model of the human Sec61α with a CRF_1_R signal peptide emerging at the lateral gate (S4C and S4D Fig). The very large dimensions of the protein conducting channel itself, however, preclude any predictions for potential FMP-401319-3 binding sites by further *in silico* approaches. Of note, the structure of FMP-401319-3 has a stretched conformational pre-orientation with a restricted degree of flexibility, which allows it to adaptively bind to an extended binding site. Although the compound is substantially smaller than a signal sequence (see S4D Fig for the dimensions), there is an option that FMP-401319-3 may bind to one or even several of the contact points in the protein-conducting channel which are normally occupied by signal sequences. We found no apparent overall similarities of our inhibitor FMP-401319-3 to other compounds known to interfere with the Sec61 complex. Nonetheless, it is interesting that Eeyrastatin 1, (see introduction) features two chlorophenyl rings, which are also present in FMP-401319-3 albeit in a very different structural context. It remains to be determined with additional structure/function studies whether this is of any significance.

Of note, whereas the number of inhibitors addressing the eukaryotic Sec61 complex has grown in the last decade [4], no specific inhibitor for the prokaryotic SecYEG complex has been reported to date (except of decatransin which inhibits both the Sec61 and SecYEG complexes [36]). The detection of specific inhibitors for the SecYEG complex by using an adapted, sequential screening assay would be extremely important since such compounds may represent antibiotics with a novel mechanism of action which are urgently needed taking the threats arising by multi drug resistant infectious bacteria into account. Although prokaryotic SecY and eukaryotic Sec61α are functionally and structurally closely related, the sequence identities and similarities are not so high which may allow the development of specific prokaryotic inhibitors (e.g. 18.7% identity and 49.6% similarity for *E*. *coli* SecY vs. human Sec61α; program LALIGN).

## Conclusion

In summary, we here established a novel sequential high throughput screening procedure that allowed the identification of novel inhibitors for the eukaryotic SRP-Sec61 targeting/translocation pathway. The potency of the final compound FMP-401319-3 is still relatively low and must be optimized in the future by medicinal chemistry once the structure/activity relationships of the compound are known in more detail. Using a much larger compound library for screening may be another approach to get compounds with higher potency. This is, however, not feasible in our academic institution. Most importantly, our screening approach may pave the way to a similar assay which may help to find inhibitors for the bacterial SecYEG complex in the future in order to detect novel antibiotic drugs.

## Supporting information

S1 Table Percentage of translocated CRF1R-pPL in the presence of different inhibitors. Signal intensities of the individual digital autoradiograms were used to determine the translocation efficiency for construct CRF1R-pPL in the cell free *in vitro* transcription/translation/translocation assay in the presence of the indicated inhibitor. The percentage translocation was calculated as the relative amount of translocated protein versus total protein (translocated and precursor) after compound treatment, in comparison to the corresponding DMSO control (= 100% translocation). Shown are the % of translocation for the individual experiments summarized in Figs 5 and 7. In addition, IC_50_ values are calculated, with mean +/- SD.
(TIF)

S1 Fig Schematic depiction of the three technical replicates of the 1052 hit compounds under selection and deselection conditions. For each compound (dots), the relative GFP fluorescence of the screening target in the primary screen (CRF_1_R.GFP) was plotted against the relative GFP fluorescence of the target in the secondary screen (soluble, unfused GFP). Hit compounds are indicated in green, the 5 compounds used for further analysis are indicated in red (see below). See Fig 2. for the calculated mean values of these 3 replicates and for % calculations.
(TIF)

S2 Fig Primary sequence and build-up of the CRF_1_R-_p_PL fusion construct. CRF_1_R-_p_PL repsents a fusion of the signal peptide (SP) of the CRF_1_R and the bovine preprolactin mature domain. For the cell free *in vitro* transcription/translation/translocation experiment, mRNAs encoding 78 residues without stop codon were used. The primary sequence is shown in the upper panel. The 78mers contain the CRF_1_R signal peptide (23 residues, red), a short CRF_1_R downstream sequence (7 residues, red) and a preprolactin sequence (48 residues, black). Methionine residues suitable for [35S] labeling are indicated in blue; the dash represents the clavage site for the signal peptidase. The lower panel shows a scheme of the construct.
(TIF)

S3 Fig The cyclodepsipetide cotransin inhibits translocation of CRF_1_R-_p_PL. Representative digital autoradiogram of the translated and translocated CRF_1_R-_p_PL chimaera in the absence or presence of different concentrations of cotransin (1 μM, 10 μM, 100 μM), similar as described in the legend to Fig 7B.
(TIF)

S4 Fig Structural homology model of human Sec61α in complex with the signal peptide of the CRF_1_R. The structure model is based on **(A)** the *Geobacillus thermodenitrificans* SecY crystal structure and **(B)** the canine Sec61α cryo-EM structure both in complex with signal peptides (OmpA, 44 amino acid residues, method X-ray diffraction, PDB entry 5EUL; and pre-prolactin, method cryo-EM, PDB entry 3JC2) respectively. Both structures are shown with an open lateral channel gate (backbone presentation). Based on these structural information, we designed **(C)** a homology model for human Sec61α with the bound signal peptide of the CRF_1_R (helical region, green) and the additional N-terminal residues from position 21 to 47. For modelling, the structures of the helical signal peptides in complex with SecY and Sec61α were superimposed and the fused amino acids from the SecY complex were introduced into the canine Sec61α (with removed pre-prolactin signal peptide). These amino acid residues were then replaced by the corresponding amino acids of the CRF_1_R from position His4 to Ser47. The resulting complex was refined by side chain minimization until converging at a termination gradient of 0.2 kcal/mol*Å with constraint backbone atoms, which were released in a second minimization step until converging at a termination gradient of 0.1 kcal/mol*Å. This first preliminary model was additionally refined by short molecular dynamic simulations (300 K, 3 ns) and energetic minimization until converging at a termination gradient of 0.1 kcal/mol*Å. Structural modifications to generate the homology models were performed with the software *Sybyl X2*.*0* (Certara, NJ, USA). For energy minimization and molecular dynamic simulations, the *AMBER F99* force field was used. A surface presentation of this complex **(D)** shows the signal peptide of the CRF_1_R embedded between helices TMH2 and TMH7 in the open lateral gate and the following amino acid residues located inside the channel. Compound 401319–3 interferes with a step before the growing peptide chain has reached the luminal side of the ER and before the signal peptide has left the lateral gate for cleavage. There are a multitude of possibilities which could explain the mechanism of action of compound FMP-401319-3: it may bind to one or even several of the contact points in the protein-conducting channel which are normally occupied by signal sequences. On the other hand, it may impair the overall helix movements inside or outside of the translocation channel which are necessary for the conversion of Sec61α from the closed to the open state. An indirect impact on the mechanism of action, however, could also not be excluded as yet. Structure images were produced using the *PyMOL* software (DeLano WL, version 1.5, San Carlos, CA, USA).
(TIF)
