# Supplementary figures and images for: Use of a sequential high throughput screening assay to identify novel inhibitors of the eukaryotic SRP-Sec61 targeting/translocation pathway
Source: PLoS One. 2018 Dec 13;13(12):e0208641. doi: 10.1371/journal.pone.0208641 (PMC6292634; doi:10.1371/journal.pone.0208641)

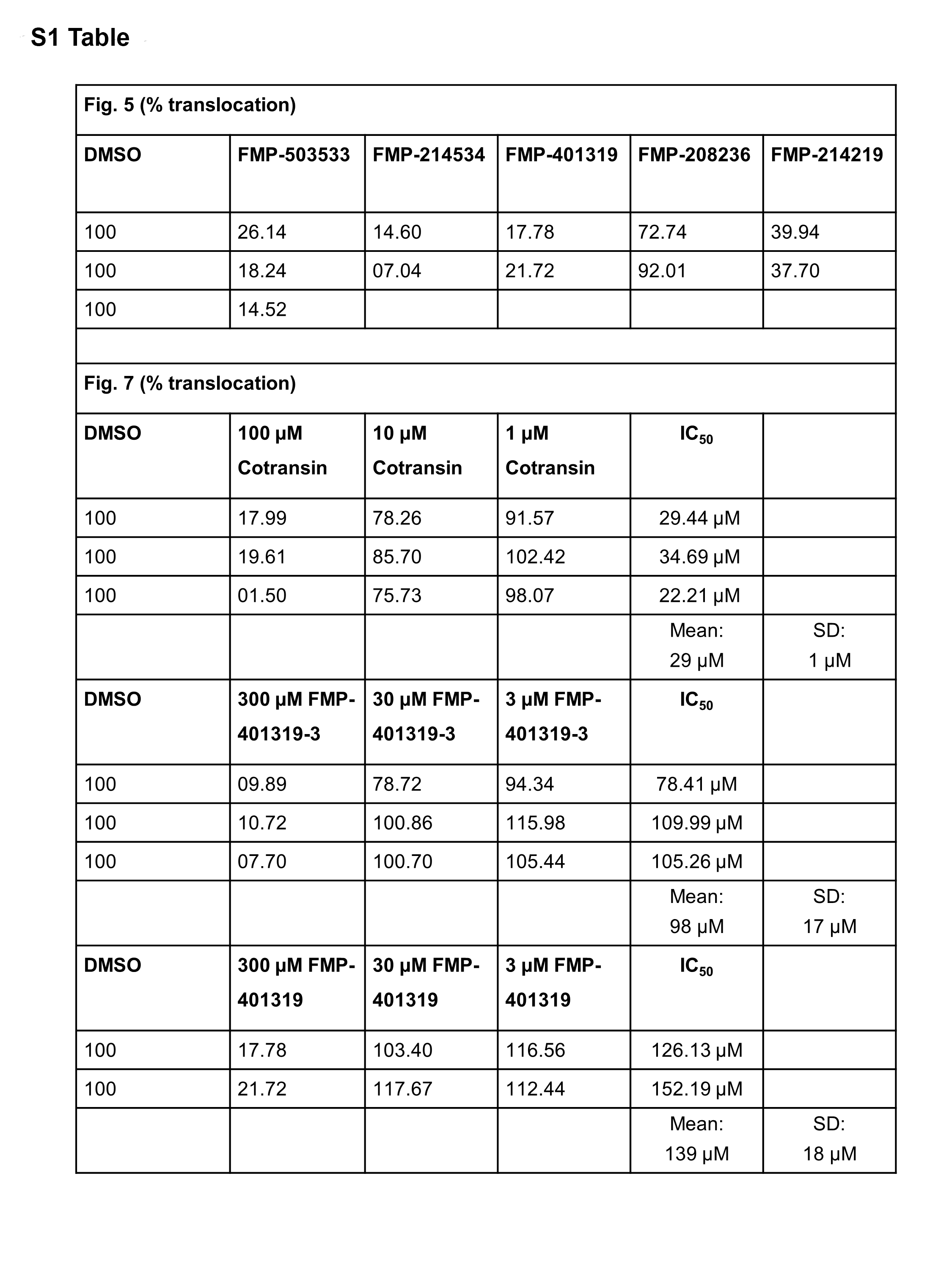

Supplement: S1 Table — Signal intensities of the individual digital autoradiograms were used to determine the translocation efficiency for construct CRF1R-pPL in the cell free in vitro transcription/translation/translocation assay in the presence of the indicated inhibitor. The percentage translocation was calculated as the relative amount of translocated protein versus total protein (translocated and precursor) after compound treatment, in comparison to the corresponding DMSO control (= 100% translocation). Shown are the % of translocation for the individual experiments summarized in Figs 5 and 7. In addition, IC50 values are calculated, with mean +/- SD. (TIF) [file pone.0208641.s001.tif]

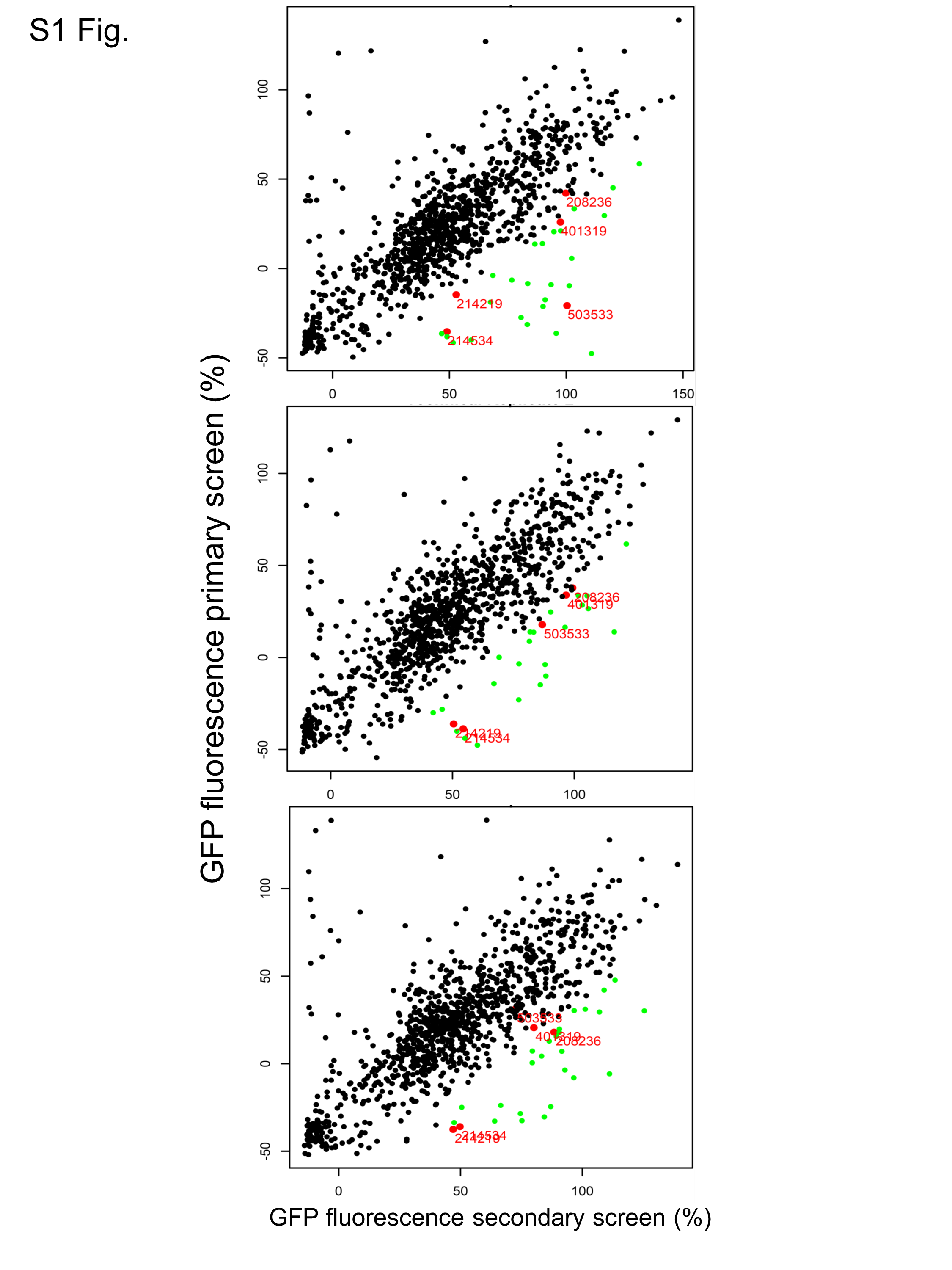

Supplement: S1 Fig — For each compound (dots), the relative GFP fluorescence of the screening target in the primary screen (CRF1R.GFP) was plotted against the relative GFP fluorescence of the target in the secondary screen (soluble, unfused GFP). Hit compounds are indicated in green, the 5 compounds used for further analysis are indicated in red (see below). See Fig 2. for the calculated mean values of these 3 replicates and for % calculations. (TIF) [file pone.0208641.s002.tif]

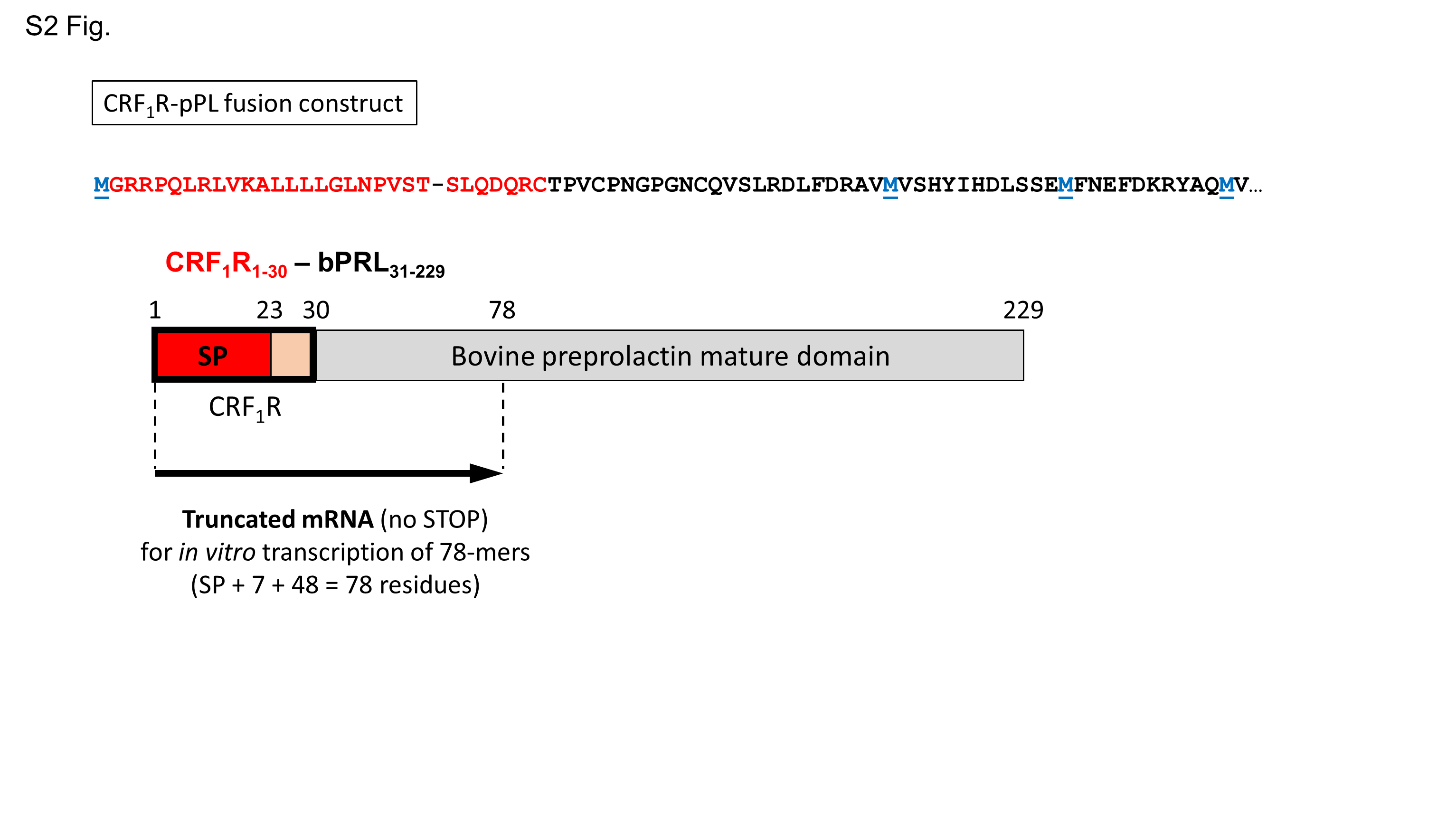

Supplement: S2 Fig — CRF1R-pPL repsents a fusion of the signal peptide (SP) of the CRF1R and the bovine preprolactin mature domain. For the cell free in vitro transcription/translation/translocation experiment, mRNAs encoding 78 residues without stop codon were used. The primary sequence is shown in the upper panel. The 78mers contain the CRF1R signal peptide (23 residues, red), a short CRF1R downstream sequence (7 residues, red) and a preprolactin sequence (48 residues, black). Methionine residues suitable for [35S] labeling are indicated in blue; the dash represents the clavage site for the signal peptidase. The lower panel shows a scheme of the construct. (TIF) [file pone.0208641.s003.tif]

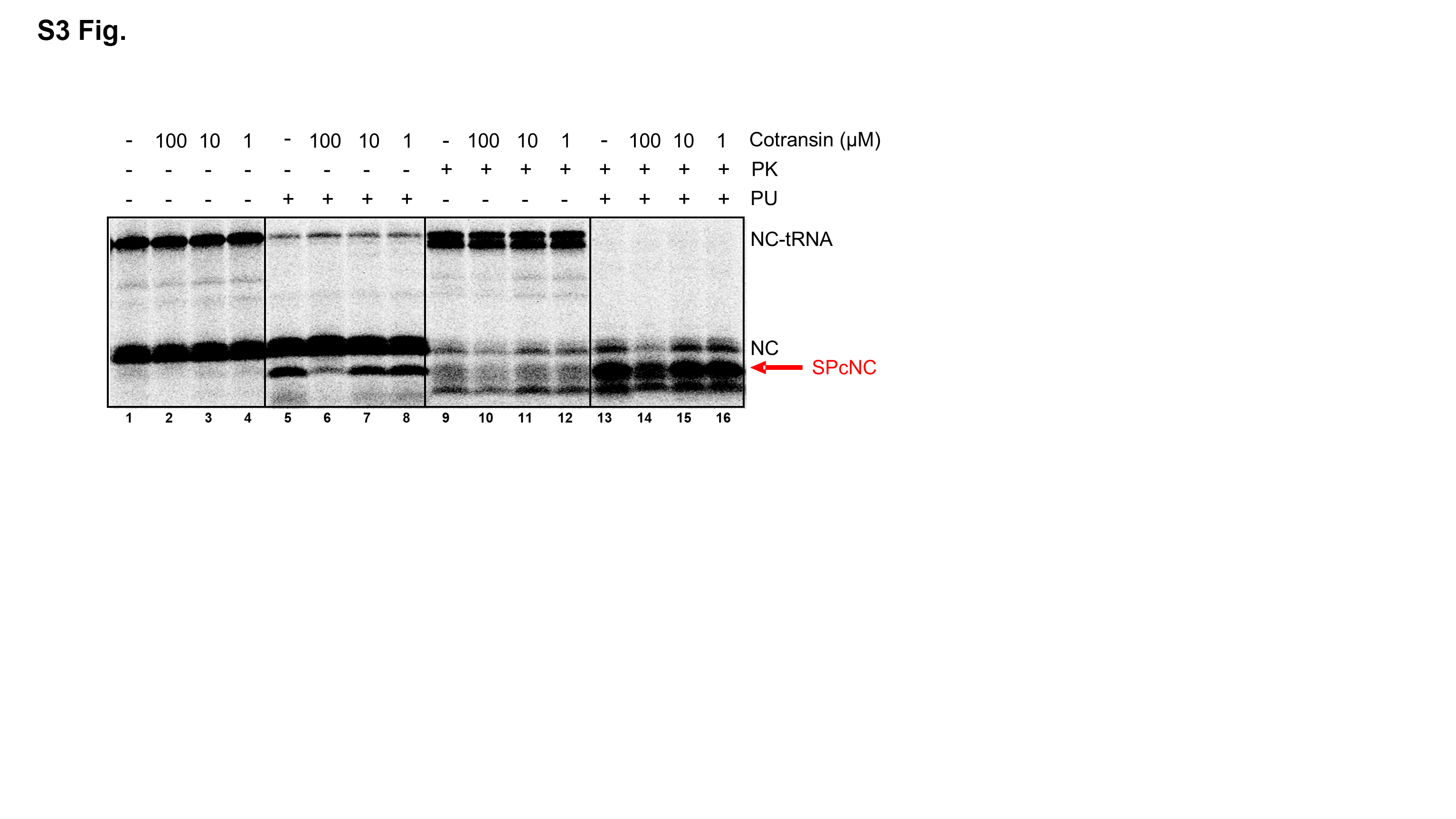

Supplement: S3 Fig — Representative digital autoradiogram of the translated and translocated CRF1R-pPL chimaera in the absence or presence of different concentrations of cotransin (1 μM, 10 μM, 100 μM), similar as described in the legend to Fig 7B. (TIF) [file pone.0208641.s004.tif]

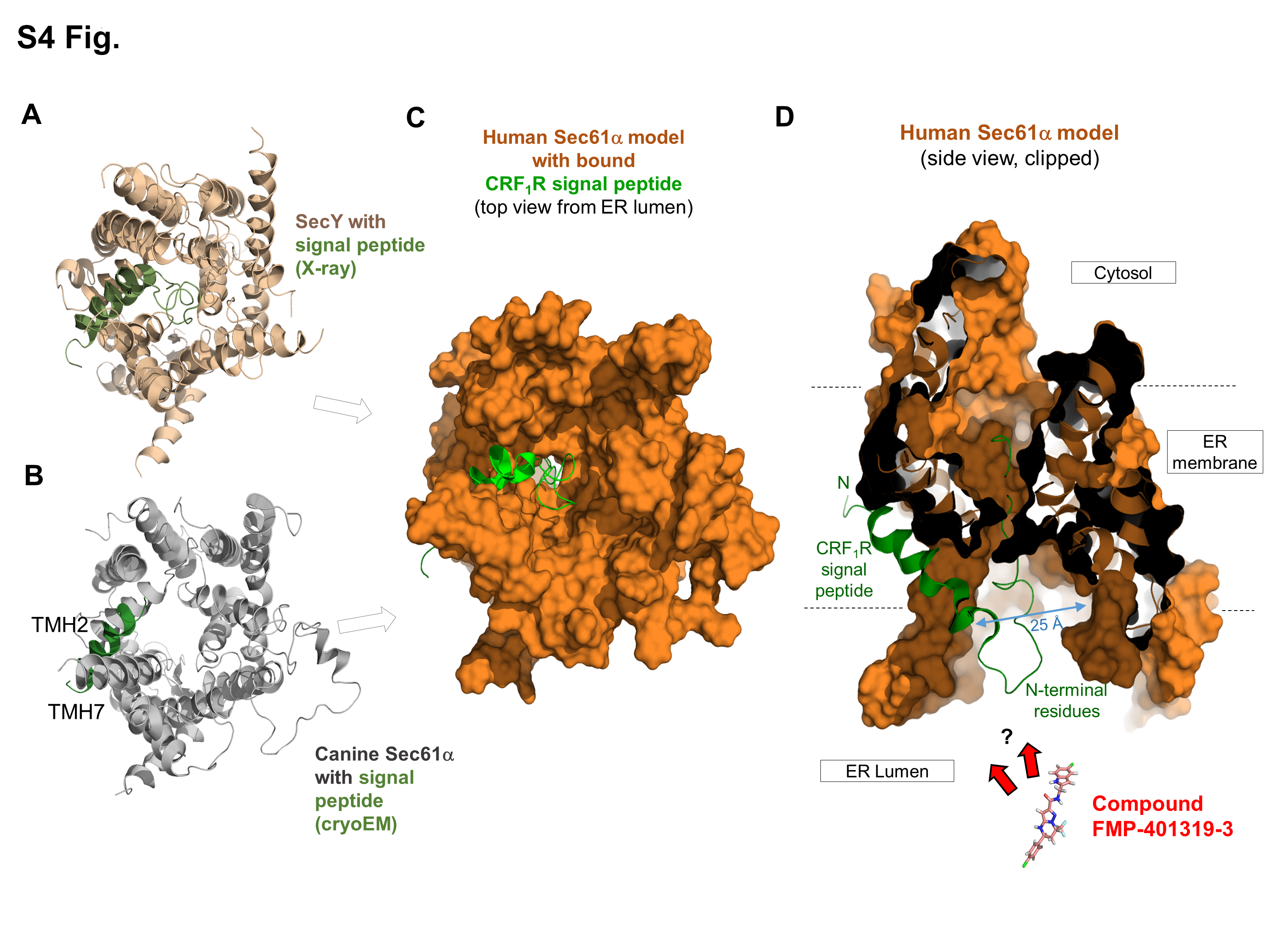

Supplement: S4 Fig — The structure model is based on (A) the Geobacillus thermodenitrificans SecY crystal structure and (B) the canine Sec61α cryo-EM structure both in complex with signal peptides (OmpA, 44 amino acid residues, method X-ray diffraction, PDB entry 5EUL; and pre-prolactin, method cryo-EM, PDB entry 3JC2) respectively. Both structures are shown with an open lateral channel gate (backbone presentation). Based on these structural information, we designed (C) a homology model for human Sec61α with the bound signal peptide of the CRF1R (helical region, green) and the additional N-terminal residues from position 21 to 47. For modelling, the structures of the helical signal peptides in complex with SecY and Sec61α were superimposed and the fused amino acids from the SecY complex were introduced into the canine Sec61α (with removed pre-prolactin signal peptide). These amino acid residues were then replaced by the corresponding amino acids of the CRF1R from position His4 to Ser47. The resulting complex was refined by side chain minimization until converging at a termination gradient of 0.2 kcal/mol*Å with constraint backbone atoms, which were released in a second minimization step until converging at a termination gradient of 0.1 kcal/mol*Å. This first preliminary model was additionally refined by short molecular dynamic simulations (300 K, 3 ns) and energetic minimization until converging at a termination gradient of 0.1 kcal/mol*Å. Structural modifications to generate the homology models were performed with the software Sybyl X2.0 (Certara, NJ, USA). For energy minimization and molecular dynamic simulations, the AMBER F99 force field was used. A surface presentation of this complex (D) shows the signal peptide of the CRF1R embedded between helices TMH2 and TMH7 in the open lateral gate and the following amino acid residues located inside the channel. Compound 401319–3 interferes with a step before the growing peptide chain has reached the luminal side of the ER and before the si [file pone.0208641.s005.tif]
